# Pigments, Parasites and Personalitiy: Towards a Unifying Role for Steroid Hormones?

**DOI:** 10.1371/journal.pone.0034281

**Published:** 2012-04-06

**Authors:** Silje Kittilsen, Ida Beitnes Johansen, Bjarne Olai Braastad, Øyvind Øverli

**Affiliations:** 1 Department of Animal and Aquacultural Sciences, Norwegian University of Life Sciences, Ås, Norway; 2 Department of Molecular Biosciences, University of Oslo, Oslo, Norway; University of Plymouth, United Kingdom

## Abstract

A surging interest in the evolution of consistent trait correlations has inspired research on pigment patterns as a correlate of behavioural syndromes, or “animal personalities”. Associations between pigmentation, physiology and health status are less investigated as potentially conserved trait clusters. In the current study, lice counts performed on farmed Atlantic salmon *Salmo salar* naturally infected with ectoparasitic sea lice *Lepeophtheirus salmonis* showed that individual fish with high incidence of black melanin-based skin spots harboured fewer female sea lice carrying egg sacs, compared to less pigmented fish. There was no significant association between pigmentation and lice at other developmental stages, suggesting that host factors associated with melanin-based pigmentation may modify ectoparasite development to a larger degree than settlement. In a subsequent laboratory experiment a strong negative correlation between skin spots and post-stress cortisol levels was revealed, with less pigmented individuals showing a more pronounced cortisol response to acute stress. The observation that lice prevalence was strongly increased on a fraction of sexually mature male salmon which occurred among the farmed fish further supports a role for steroid hormones as mediators of reduced parasite resistance. The data presented here propose steroid hormones as a proximate cause for the association between melanin-based pigmentation and parasites. Possible fundamental and applied implications are discussed.

## Introduction

Intraspecific variation in animal pigmentation has spurred many of the most active and exciting fields in evolutionary biology such as sexual selection, sympatric speciation, crypsis and mimicry. Most fascinating is perhaps the fact that alternative colour morphs within a species also often differ in other traits than colour. Recent interest in the evolution of consistent trait correlations has inspired research on pigmentation as a correlate of behavioural syndromes, or “animal personalities” [1], [2]. Colour polymorphisms could be very useful in this context, because they are easily recognised and provide information about other traits without extensive physiological, behavioural and genetic analysis. However, in a majority of cases, a much stronger interest has been directed towards the theoretical basis for apparent co-selection, than towards causative molecular-genetic and physiological mechanisms which actually mediate the links.

In vertebrates, two main pigment groups - carotenoids and melanins - cause colour variation. Variation in carotenoid pigmentation is mostly condition-dependent and is assumed to signal individual “quality”, in the sense that carotenoids are in limited supply, and also have other critical functions than ornamentation, e.g. in antioxidative processes and immune responses [3], [4], [5], [6]. Stable colour polymorphisms, on the other hand, often involve genes responsible for synthesis and deposition of melanins - in vertebrates the black pigment eumelanin, and red to brownish phaeomelanin [2], [7].

Melanin-based plumage colouration has in several bird species been associated with not only specific behavioural patterns (reviewed by Ducrest et al. [1]), but also the magnitude of the immune response and resistance to ectoparasites [8]–[10]. Furthermore, an association between melanin-based pigmentation and behavioural-physiological aspects of animal personality appears to be widespread in the vertebrate subphylum [1], [11]–[16], with more distinctly pigmented individuals typically being more proactive, stress-resistant, and socially dominant. In salmonid fishes (which produce only the black pigment eumelanin) melanin-based colour polymorphisms are evident as distinct black skin spots, and more melanised individuals show a reduced physiological and behavioural response to stress [14]. Melanin-based colour patterns are to some extent condition dependent [12], [17], [18], but the above trait correlation is clearly to a large degree genetically determined: Selection studies in rainbow trout (*Oncorhynchus mykiss*) aimed at one trait only – divergent post-stress cortisol levels [19] – has yielded high- (HR) and low-responsive (LR) strains which differ in both physiology [19], [20], behaviour [21]–[25], and eumelanin-based pigmentation [14]. Through their body-wide coordinating effects on gene expression, as well as through more recently discovered non-genomic receptors, steroid hormones such as corticosteroids and sex steroids play a central role in control of behaviour and personality traits [24], [26]–[29]. As an example, many behavioural features of the HR rainbow trout line can be mimicked by exogenous cortisol [30], [31].

In addition to activational and organisational effects on behaviour, steroids are important modulators of the immune system [32], [33], including those components involved in parasite defence [34]. Parasites may be a major selective force in determining the fitness of variable melanin morphs [35], and strong effects of ectoparasites on fish population biology have been reported in several studies [36]–[38]. Recently, several authors have also argued that parasites may be important agents in the evolution of animal personalities [39], [40]. The potential role for steroid hormones as key mediators of parasite resistance in concert with other personality traits has however not been widely apprehended in the literature. One question which has not been investigated is whether a positive relationship between parasite resistance and melanin pigmentation is present in the otherwise well studied salmonid model system. Revealing whether such an association is present in fishes could potentially provide new cues to both fundamental, conserved mechanisms, and more applied implications of an intriguing multi-trait correlation.

In the following we address this question in Atlantic salmon (*Salmo salar*). Farmed salmon held at high densities provide a favourable environment for parasites (e.g. ectoparasitic salmon lice, *Lepeophtheirus salmonis*) [41], and the large number of hosts held in a homogenous environment produces a tenable opportunity to investigate individual variation in parasite susceptibility. Colour polymorphisms are clearly evident, as illustrated by the black eumelanin-based skin spots on salmonid fishes [14]. Additionally, a link between melanin-based pigmentation and stress-induced cortisol production has been demonstrated in juvenile fish. It is however not known whether this association is disrupted by the transition from freshwater to saltwater which occurs in both wild and farmed anadromous salmonids, during which cortisol is critically involved in osmoregulatory adaptation and energy metabolism [42], [43]. Thus, in addition to testing the hypothesis that less pigmented fish carry higher loads of *L. salmonis*, a supplementary objective of this study was to investigate whether a relationship between pigment patterns and the corticosteroid response to stress is present in adult Atlantic salmon.

## Materials and Methods

### Ethics statement

All experimental procedures complied with Norwegian ethical standards for research involving animals. Lice counts were performed in line with prescribed lice surveillance routines (http://www.lovdata.no/for/sf/fi/fi-20090818-1095.html), which does not require approval by an ethics committee. The acute stress experiment was reviewed and approved by the Norwegian Animal Research Authority (*Forsøksdyrutvalget*; www.fdu.no).

### Experimental fish and locations

Lice registrations and quantification of skin melanisation were performed on n = 48 adult Atlantic salmon (body mass range 2.9–6.8 kg) naturally infected with lice at the Marine Harvest (www.marineharvest.no) seawater site in Lysøysund, Norway (63°41′N; 9°39′E). Logistics of sampling, which was performed from a service boat, dictated that individual fish were captured from two different sea nets containing two different domesticated populations (MOWI n = 23 and Aquagen “Robust” n = 25). Only non-invasive observations could be performed on the adult net-reared salmon, which were part of a multi-generation breeding programme. Experimental fish used for stress-testing were therefore commercially available Atlantic salmon (n = 19) in the seawater on-growth phase (485±105 g; Mean ± SD) kept in seawater in a 1000 l circular indoor tank at Solbergstrand Research Station (www.niva.no). The tank had continuous water supply (8–10°C), and the fish were fed approximately 1.5% of the fish biomass daily 5 days a week.

### Quantification of melanin-based pigmentation

Following Kittilsen et al. [14], melanin-based skin spots were counted in a defined area above the sideline, reaching from the gill cover to the base of the dorsal fin on both sides of each fish. The use of measuring tape with millimetre resolution, allowed for an accurate estimation of this area. Fish body mass was estimated from the dimensions of this area using a previously determined formula, approximating a linear relationship between skin area and body mass in the relevant size segment. A single observer assigned skin spots into the size categories “small”, “medium” and “large” corresponding to a numerical factor of 1, 1.5, and 2, with which the counted number of spots in each size category was multiplied. This was done to get an approximate measure of the total amount of visible melanin. Individual values for the left and right side were averaged and the degree of melanin-based coloration was expressed as % of population mean.

### Lice registrations

The salmon louse has a life cycle of 10 stages, where eight of them are parasitic on the host fish. Mature female lice carry fertilized eggs in pairs of egg sacs containing up to 1000 eggs. Fish were caught randomly from the sea nets and transferred to a white container containing approximately 100 l of sea water and a small amount of sedative (100 mg/l MS-222). The whole body of the fish was screened and both the number of mature female lice and the number of lice at other developmental stages were registered, where after skin melanisation was quantified as above. A small group of sexually mature male fish were recognised based on secondary sexual characteristics and running milt (n = 5 out of n = 48 total), and was treated separately in the statistical analysis.

### Stress-testing

The cortisol response in individual fish was investigated by an acute confinement test, corresponding to tests previously used to investigate heritability of the stress response [19] and to describe contrasting stress coping styles in rainbow trout and salmon [14], [24], [44]. Tested individuals were subjected to confinement in a transparent plastic chamber with 5 l aerated water. At 30 min following the onset of stress, the fish were removed from the chambers and anaesthetized with MS-222 (250 mg/l). Within two minutes after capture, blood samples were drawn from the caudal vein using heparinized 1 ml syringes, where after skin melanisation was quantified as above. The blood samples were kept on ice until centrifuged for 5 minutes at 10 000 rpm. Plasma was decanted off and frozen at −80°C until analyses of plasma cortisol content. Plasma cortisol concentrations were quantified using a radioimmunoassay (RIA) according to the protocol of Pottinger & Carrick [21]. All samples were analysed in duplicates in a single assay, and the intra-assay coefficient of variation was 5.1%. The antibody used was Abcam@1∶1500 and the lower detection limit for the assay was 0.3 ng/ml. Cross-reactivity of the antibody with cortisone, the most abundant potential competitor in salmon plasma, was 2.6%.

### Statistical analysis

Two parameters for lice densities were analysed; numbers of egg-carrying female lice and numbers of lice (either male or female) at other developmental stages per individual fish. Lice numbers did not differ between the sampled populations (mean ± S.E., egg-carrying females: 2.2±0.5 vs. 2.7±0.4; other lice: 12.5±1.7 vs. 12.1±0.8). Data from the two sampled sea nets were therefore pooled, in the sense that both fish size and skin melanisation was normalised and presented as % of population mean, prior to regression analysis. After normalisation against population averages, the relationship between skin melanisation and lice numbers followed a single regression line (ANCOVA, data not shown), with deviation from linearity assessed by runs test and statistical strength assessed by linear regression analysis. The relationship between skin melanisation and post-stress plasma cortisol levels was also confirmed statistically by linear regression analysis. An effect of sexual maturation on parasite abundance was confirmed by non-parametric Mann-Whitney U-test (due to lack of variance homogeneity between mature and immature fish), while t-test was used to analyse whether early sexual maturation was associated with a difference in pigmentation or body mass (variance homogeneity confirmed by Levene's test, Gaussian distributions confirmed by Kolmogorov-Smirnov method).

## Results

### Melanin-based pigmentation and lice prevalence

**Figure 1:**
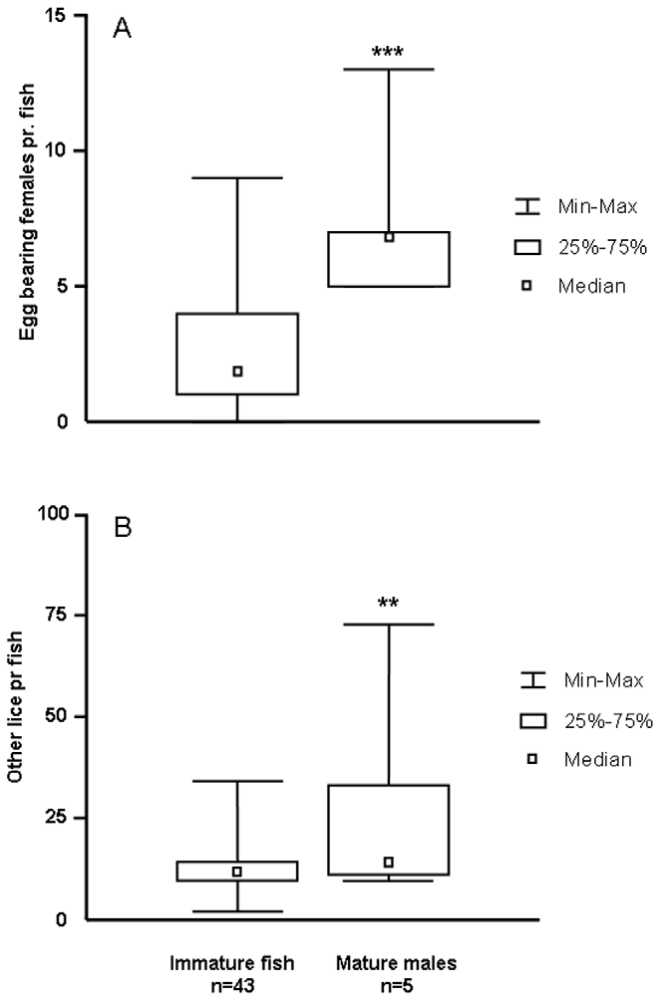
Lice counts in immature fish (both sexes, n = 43) and precociously mature males (n = 5), A) Counts of mature female lice carrying egg sacs B) Numbers of lice (male and female) at other developmental stages per fish, ** = p<0.01, *** = p<0.001, Mann-Whitney U-test.

**Figure 2:**
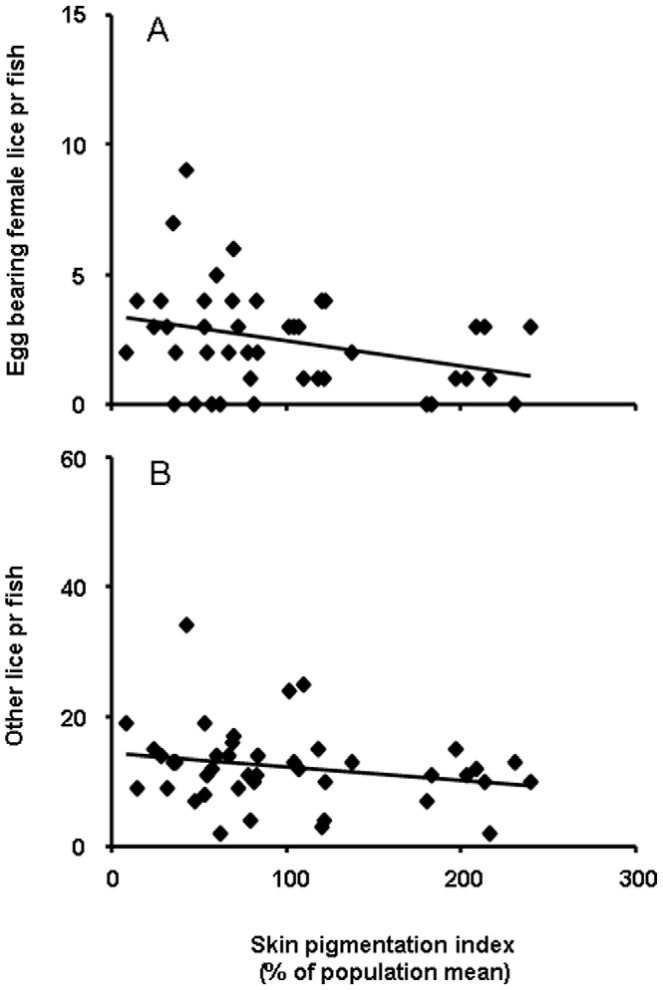
Linear regression between skin melanisation and lice counts in reproductively immature Atlantic salmon reared in seawater. A) Counts of mature female lice carrying egg sacs (r^2^ = 0.10, p = 0.04). B) Numbers of lice at other developmental stages (R^2^ = 0.05, p = 0.15).

**Figure 3:**
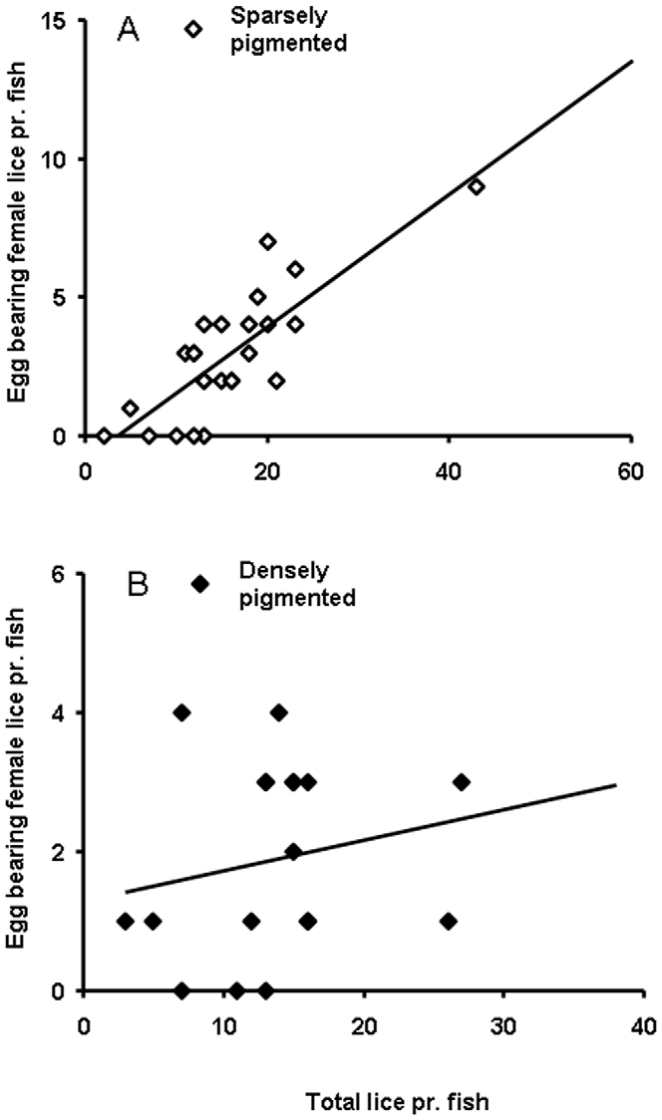
Numbers of mature female lice carrying egg sacs as a function of total lice on each fish. Individuals with different degrees of melanin-based skin pigmentation were categorised with respect to population average. A) Fish with below average pigmentation (“sparsely pigmented”, R^2^ = 0.66, p<0.001). B) Fish with above average pigmentation (“densely pigmented”, R^2^ = 0.04, p = 0.43).

Based on the presence of running milt and spawning colours, 5 out of 48 fish were identified as precociously mature males. These fish carried significantly higher parasite loads than the remainder of the sampled individuals, both with regards to egg-bearing females (Mann-Whitney U-test, p<0.001, figure 1A) and other lice (Mann-Whitney U-test, p = 0.004, figure 1B). Sexually mature males were significantly smaller than other fish (estimated body mass 3667±217 g vs. 4954±109 g, mean ± S.E., t_(46)_ = 3.9, p<0.001), but there was no relationship between fish body mass and parasite burden in this subset of fish (egg bearing females: r^2^ = 0.14, p = 0.53; other lice: r^2^ = 0.24, p = 0.40) or in the remainder of the study material (egg bearing females: r^2^ = 0.03, p = 0.30; other lice r^2^<0.001, p = 0.91). There was no effect of sexual maturation on skin melanisation (t_(46)_ = 0.40, p = 0.69), nor any significant relationship between pigmentation and parasite burden in this group (egg bearing females: r^2^ = 0.17, p = 0.48; other lice r^2^ = 0.30, p = 0.35). In immature fish, there was a significant negative association between melanin pigmentation and numbers of mature female lice carrying egg sacs on each individual fish (R^2^ = 0.10, p = 0.04, figure 2A). This relationship was not statistically significant when only other lice were considered (R^2^ = 0.05, p = 0.15, figure 2B). Thus it would appear that the most melanised fish in the population were able to some degree modify the presence of egg-bearing females, but not infection by lice as such. To further examine this effect, we studied the number of egg-bearing females as a function of total lice. For this purpose, the sample material was divided in two categories with respect to the population mean, “densely pigmented” (all fish expressing above average melanisation) and “sparsely pigmented” (all fish expressing below average melanisation). This approach revealed that in the sparsely pigmented group, the number of egg bearing females co-varied strongly with the total number of lice present (linear regression; r^2^ = 0.66, p<0.001, figure 3A), while no such relationship was indicated in the densely pigmented group (figure 3B).

### Melanin- pigmentation and post-stress cortisol

**Figure 4:**
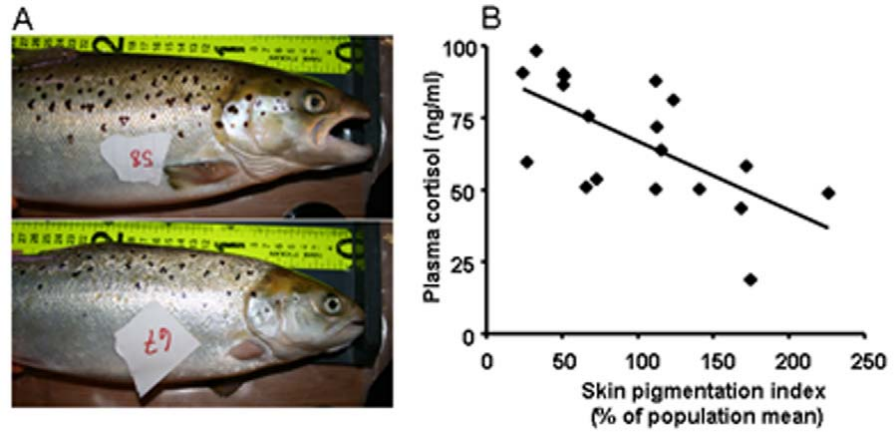
Relationship between melanin-based pigmentation and the cortisol response to stress in Atlantic salmon kept in seawater. A) Photos taken during the stress-test experiment showing representative phenotypic variation in melanin-based skin spots. B) Regression between melanin-based skin pigmentation and post-stress plasma cortisol levels (R^2^ = 0.66, p = 0.003).

Figure 4A shows two representative pictures from the stress experiment, illustrating variable skin pigmentation in Atlantic salmon reared in seawater. Acute stress induced by confinement revealed a negative correlation between skin melanisation and post-stress plasma cortisol concentrations in adult Atlantic salmon (R^2^ = 0.66, p = 0.003, Fig. 4B), i.e. more spotted individuals showed a reduced cortisol response to acute stress compared to less spotted individuals.

## Discussion

Physiological and environmentally induced colour changes are well documented in fishes [45], [46], but more permanent colour polymorphisms are little investigated in this animal group. The data presented above confirm that the degree of melanin-based skin pigmentation, expressed as black skin spots, is associated with at least two other distinct phenotypic traits in Atlantic salmon: Resistance to ectoparasitic salmon lice, and magnitude of the cortisol response to acute stress. In the current study we did not investigate baseline levels of cortisol production, but unless farmed Atlantic salmon live their entire life in a stress-free environment, it is safe to assume that less pigmented individuals are over time exposed to higher levels of corticosteroid hormones. Thus, steroid hormone exposure likely constitutes an important, but largely overlooked, proximate mechanism in the often observed association between melanin-based pigmentation and other fitness related traits [8], [47]–[50].

It may be argued that although steroid suppression of immune function has been observed throughout various vertebrate taxa [32], [51], other properties of melanin pigments may be important specifically in host-parasite interactions. Due to their physical and chemical properties, melanins protect against physical damage and infectious agents, UV-light, and toxicants, among other things [52]–[54]. Insects protect themselves by internalizing parasites in melanin [55], [56], and so do at least some fishes [57]. However, the fact that reproductively mature male Atlantic salmon showed a dramatic increase in lice numbers, but no change in melanin-based pigmentation, strongly suggest steroid-induced immunosuppression as a proximate mechanism. Like other salmonid fishes, Atlantic salmon show large variability in age and size at puberty, both between and within strains [58]–[60]. Levels of both sex hormones and cortisol increase during sexual maturation in salmonids [61]–[64], and effects of these hormonal changes on immune function are well documented (see e.g. reviews by Harris and Bird [65]; Taranger et al. [66]). There was no indication that skin melanisation differed between mature and immature fish in our sampling material, leaving steroid hormones as a likely causal agent for decreased parasite resistance. Indeed, it has previously been shown that treatment with exogenous cortisol will enhance susceptibility to infection by *L. salmonis* [67]. It however remains unknown whether cortisol directly inhibits eumelanin production, or whether other factors potentially affecting both hormonal responses and pigmentation causes this association [1].

The life cycle of *L. salmonis* encompasses ten stages: two nauplii, a copepodid, four chalimus, two pre-adult, and an adult stage [68]. Mature females can be recognised by a pair of uniseriate egg strings containing up to 1000 eggs. After hatching, free swimming nauplii and copepodid larval stages utilize nutrient reserves provided maternally. The infectious copepodids has an ability to settle on and recognize relevant hosts using a variety of cues (reviewed by Costello [41]). Chalimus stages are sessile and physically attached to the host by a frontal filament, while adult and pre-adult stages are mobile and can to some degree move between hosts. Host factors can affect both lice settlement and development [69], [70], as evidenced by differences in host susceptibility among different salmonid species (e.g. [71]–[73]. Johnson and Albright [71] for instance reported that coho salmon (*Oncorhynchus kisutch*) produce a inflammatory reaction which expels parasites during chalimii stages. Atlantic salmon, on the other hand, show enhanced expression of immune-related genes following initial infection with *L. salmonis* [74]–[76] but this immunological stimulation appears ineffective to reduce numbers of attached lice larvae. Intraspecifc differences also exist, with respect to both infectivity [77] and parasite developmental rates [78].

Our data revealed that in immature fish variation in melanin-based skin pigmentation was significantly correlated to counts of mature female lice carrying egg strings, but numbers of lice at other stages were not significantly associated with pigmentation. Mature males, on the other hand, showed increased prevalence of both egg-carrying female lice and other *L. salmonis*. Hence, in the case of variable melanisation and stress resistance, host factors would appear to affect parasite development to a larger degree than settlement. Another alternative, that female *L. salmonis* at early life stages has an ability to avoid unsuitable hosts, can not be ruled out since we could not distinguish between early developmental stages of male and female *L. salmonis* in this field study.

The above observations raise a number of interesting questions. Sea lice are considered a potential ecological and economical calamity in connection with the rapidly expanding salmon aquaculture industry. Pinpointing the biological differences between susceptible and resistant hosts may aid the development of sustainable forms of parasite control, as current chemotherapeutant treatments against salmon lice not only raise environmental concerns, but their efficacy is also decreasing [79], [80]. In addition to the potential applied aspects, from a fundamental point of view it would be of interest to explore proximate mechanisms of parasite resistance along with the ecological and evolutionary processes which maintain variability in this trait. The fact that mature males harbour more parasites is well in line with the immunocompetence handicap theory [81]. This theory accounts for possible advantages and disadvantages of high androgen levels. But what can be the advantage of high production of corticosteroid hormones? The HR-LR selection regime in rainbow trout, which originally revealed the relationship between skin pigmentation and stress coping style [14], provides a tenable hypothesis here. Low-responsive (LR) rainbow trout are generally more proactive and become socially dominant over HR fish [21]; see also reviews by Øverli et al. [24] and Schjolden and Winberg [23]. There is, however, a notable exception to this pattern: Non-pigmented HR fish compete successfully against LR fish after transport accompanied by a period of starvation [82]. The hypothesis emerges that alternative behavioural strategies reflect adaptation to different levels of environmental richness, in terms of nutritional resources. This idea is partly supported by studies on birds [18].

Further to the above, it has now been shown that melanin-based pigmentation correlate not only with behavior and physiology, but also with immunocompetence and resistance to ectoparasites in both birds [7], [8] and fishes. There is also some evidence that nutritionally rich environments sustain higher levels of infectious agents [83]–[85]. Thus, animal personality and correlated variability in parasite resistance may in fact reflect a trade-off invoked by fluctuating levels of infectious agents and nutritional resources, enabling animals to invest energy in defence systems according to available resources and need. Very likely, variable levels of steroid hormones serve to attune energetically costly immune responses to expected demand both directly and through genetically linked control mechanisms. The emerging connection between pigments, parasites and personality implies that at least some species visually and continuously signal which strategy they follow in this respect.
